# Reversible atrial fibrillation secondary to a mega-oesophagus

**DOI:** 10.1186/1472-6815-6-15

**Published:** 2006-12-13

**Authors:** Tahwinder Upile, Waseem Jerjes, Mohammed El Maaytah, Sandeep Singh, Colin Hopper, Jaspal Mahil

**Affiliations:** 1 Oral & Maxillofacial Surgery/Head & Neck Unit, University College London Hospitals, London, UK; 2 General Practice, University College London Hospital, London, UK

## Abstract

**Background:**

Atrial fibrillation (AF) is the most common cardiac arrhythmia, and it increases in prevalence with advancing age to about 5% in people older than 65 years.

**Case presentation:**

We present a rare case of atrial fibrillation secondary to a mega-oesophagus occurring in an 84-years-old Caucasian woman. The patient had a history of progressive dysphagia and the accumulation of food debris lead to mega-oesophagus.

**Conclusion:**

The diagnosis was made by barium swallow and electrocardiogram; evacuations of 300 ml of the food debris lead to complete resolution of the arrhythmia. The possible aetiology leading to this AF is discussed.

## Background

Atrial fibrillation (AF) is the most common cardiac arrhythmia, and it increases in prevalence with advancing age to about 5% in people older than 65 years (Table 1). The chance of developing atrial fibrillation at age 40 years or older is about 25% in men and women. This arrhythmia accounts for about one-third of all strokes, and 30% of all patients with atrial fibrillation have a family history of the disease [1].

**Table 1:** Causes of Atrial Fibrillation

**Atrial Fibrillation**	
**Common causes**	Heart failure
	Hypertension
	Cardiac ischemia
	Myocardial infarction
	Mitral valve disease
	Pneumonia
	Hyperthyroidism
	Alcohol
	Postoperative AF
	
**Rare causes**	Cardiomyopathy
	Constrictive pericarditis
	Sick sinus syndrome
	Bronchial carcinoma
	Atrial myxoma
	Endocarditis
	Haemochromatosis
	Sarcoidosis

When the atria are in fibrillation, contraction occurs at rates of 350–900 per minute. The AV node may conduct these impulses to the ventricles at 90–170 beats per minute, and often higher. There are several complementary and competing theories regarding the pathophysiology of AF initiation and propagation. The occasional impulses conducted by the atrio-ventricular node results in a totally irregular ventricular rhythm which is a characteristic of the condition which can be either continuous (acute or chronic) or paroxysmal [2].

Achalasia is primarily associated with a degeneration of ganglion cells of Auerbach's plexus resulting in an absence of oesophageal peristalsis and failure of lower oesophageal sphincter relaxation. This results in oesophageal dilatation or mega-oesophagus [3].

We present a rare case of a patient with achalasia in which accumulation of undigested food lead to mega-oesophagus and atrial fibrillation.

## Case presentation

An 84-year-old Caucasian women referred by her General Practitioner complaining of dysphagia and dyspnoea exacerbated by swallowing. She reported a 3 week history of progressive dysphagia, initially for solids and subsequently for fluids leading to total dysphagia. Other reported symptoms included regurgitation, cough and hoarseness of voice. There was no significant medical, family or social history.

Clinical examination revealed a rapid, irregularly irregular pulse; no other cardiovascular-respiratory abnormalities were identified. Abdominal examination also revealed no abnormality; indirect laryngoscopy which was carried out by the same examining clinician showed pooling of saliva in the hypopharyngx.

**Figure 1:**
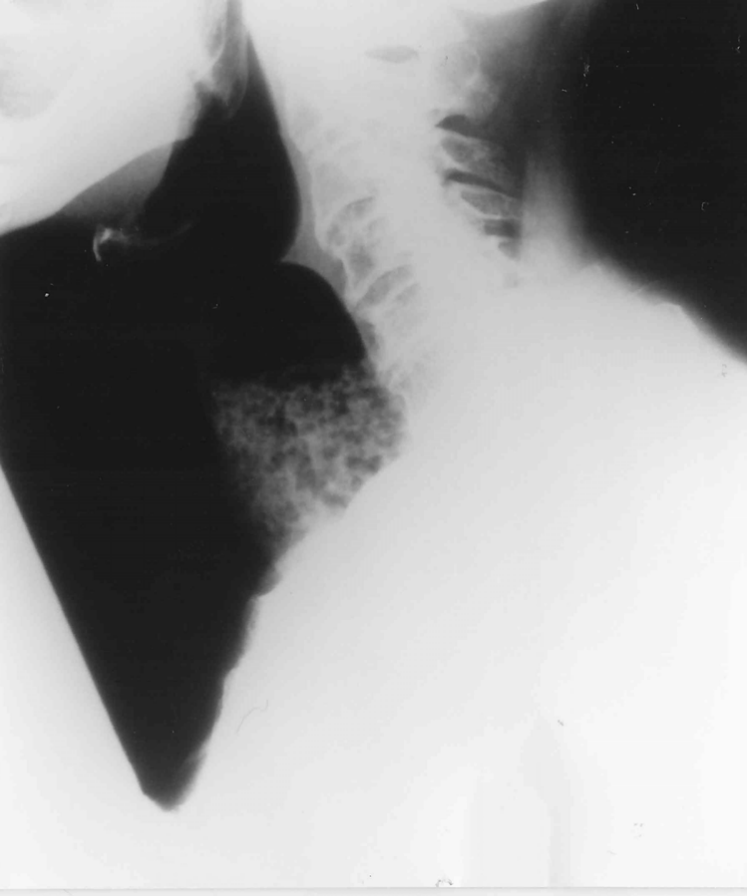
Lateral soft tissues X-ray revealing undigested material in the cervical oesophagus.

**Figure 2:**
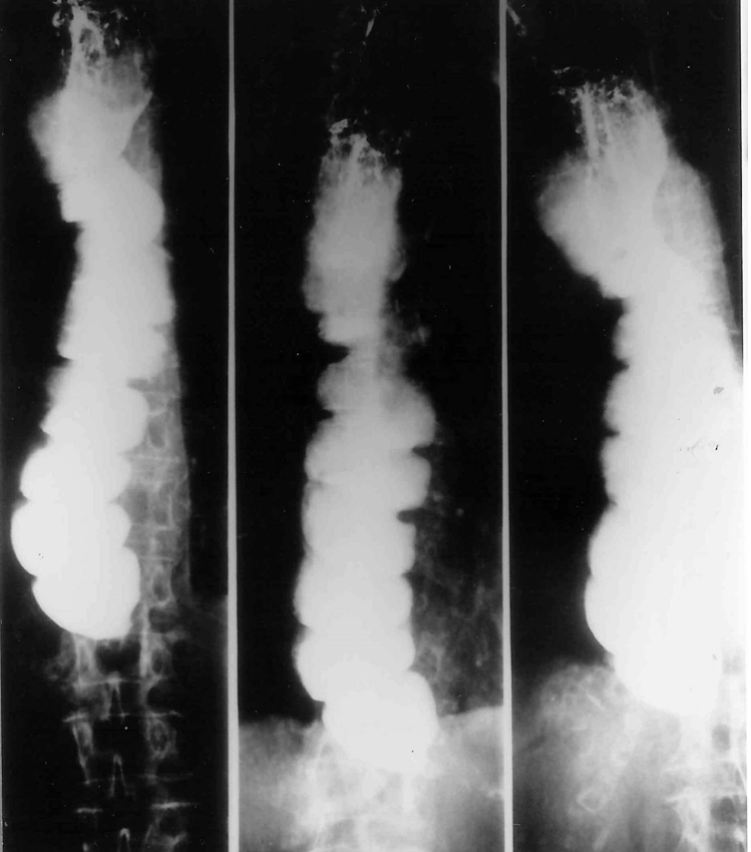
Barium meal study suggesting achalasia.

**Figure 3:**
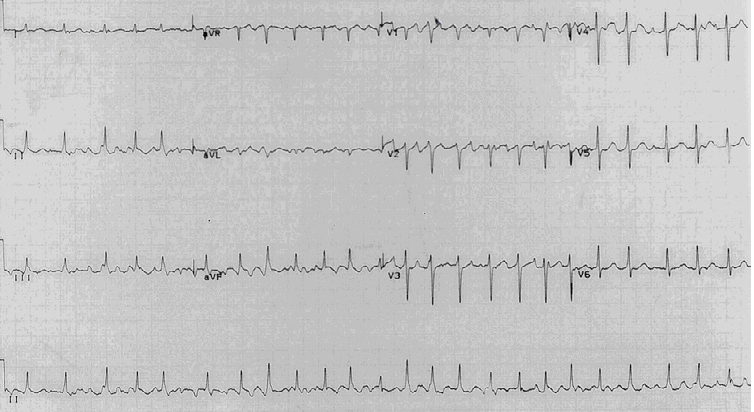
Pre-operative electrocardiogram showing atrial fibrillation with a rapid ventricular response rate.

Lateral soft tissue radiograph (Figure 1) revealed widening of the upper mediastinum and subsequent barium swallow (Figure 2) showed achalasia with food debris filling the cervical oesophagus. An electrocardiogram (ECG) showed evidence of ischaemia and atrial fibrillation with a ventricular response rate of 120–150 (Figure 3). Haematological and biochemical laboratory investigations were within the normal limits

**Figure 4:**
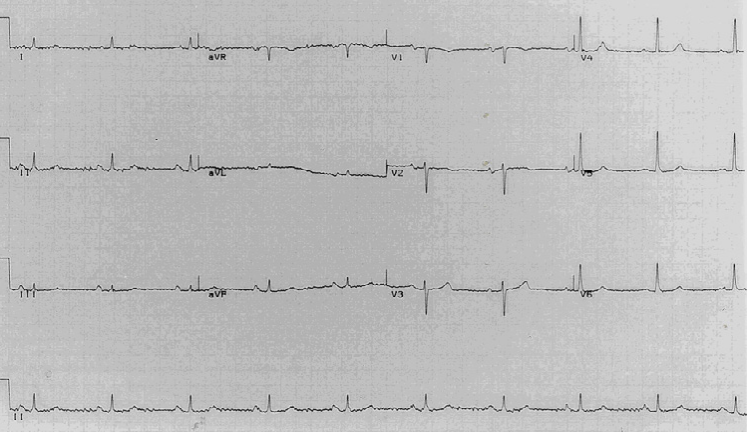
Immediate post-operative electrocardiogram showing reversion to sinus rhythm.

The patient was initially resuscitated with intravenous fluids, followed by naso-oesophageal leverage and removal of 300 ml of retained food debris from the cervical oesophagus. An ECG performed 8 hours afterwards revealed reversion back to sinus rhythm (Figure 4). Eventually the patient underwent fibre optic balloon dilatation of the distal oesophageal stricture with multiple biopsies which revealed no malignancy. Following the procedure the patient remained asymptomatic.

## Conclusion

There is a correlation between achalasia and cardiac arrhythmia [4]. Many patients with achalasia are known to perform a temporary valsalva manoeuvre to aid the transit of food over the functionally stenotic segment of their oesophagus. The anatomical proximity of the oesophagus with the left atria is further evidenced by the rare but severe complication of left atrial radiofrequency ablation of oesophageal perforation and mediastinitis [5].

Using 3 non-invasive tests, abnormalities of cardiovascular reflex function were found in 7 of 15 patients with achalasia. Abnormalities of heart rate responses to the valsalva manoeuvre, deep breathing, and standing were noted in patients with autonomic neuropathy defect. The findings are consistent with the hypothesis that an abnormality of vagal function may contribute to the pathogenesis of achalasia [6].

Achalasia itself may be a result of an abnormality of vagal function due to an autonomic neuropathic defect and therefore abnormalities of cardiovascular response are not uncommon [7].

In this patient, the removal of the food debris from the cervical oesophagus caused reversion of this arrhythmia back into sinus rhythm. This can be justified as either due to vagal repression or the relief of the pressure effect of the volume of the food debris transmitted directly to the atria. The evacuation may have resulted in either a sinus massage or a forced valsalva leading to reversion of the rhythm.

There are several confounding factors as to the exact cause of the transient atrial arrhythmia in this patient. Previous electrocardiograms exhibit a degree of ischaemia which may predispose to transient arrhythmias in situations of vagal repression especially in the circumstances of extra-cardiac intra-thoracic pathology as in this case.

## Competing interests

The author(s) declare that they have no competing interests.

## Authors' contributions

**TU**: designed the study, carried out the literature research, clinical study and manuscript preparation.

**WJ**: carried out the literature research, manuscript preparation, and manuscript review.

**ME**: carried out the manuscript editing and manuscript review.

**SS**: carried out the manuscript editing and manuscript review.

**CH**: carried out the manuscript editing and manuscript review.

**JM**: designed the study, carried out the literature research, clinical study and manuscript preparation.

All authors read and approved the final manuscript.

## Pre-publication history

The pre-publication history for this paper can be accessed here:


